# The FOXO3-FOXM1 axis: A key cancer drug target and a modulator of cancer drug resistance

**DOI:** 10.1016/j.semcancer.2017.11.018

**Published:** 2018-06

**Authors:** Shang Yao, Lavender Yuen-Nam Fan, Eric Wing-Fai Lam

**Affiliations:** Department of Surgery and Cancer, Imperial College London, Hammersmith Hospital Campus, London W12 0NN, UK

**Keywords:** ABCB1, ATP-binding cassette sub-family B member 1, AML, acute myeloid leukaemia, AMPK, adenosine monophosphate-activated protein kinase, ATM, Ataxia-telangiectasia mutated, AURKA, aurora kinase A, BER, base excision repair, Bim, B-cell lymphoma 2 interacting mediator, BRCA1, breast cancer susceptibility gene product 1, BRCA2, breast cancer-associated gene 2, BRIP1, BRCA1-interacting protein-terminal helicase 1, ChIP-seq, chromatin immunoprecipitation with massively parallel DNA sequencing, Cks1, cyclin-dependent kinases regulatory subunit 1, CML, chronic myeloid leukaemia, CSCs, cancer stem cells, DDR, DNA damage response, DSBs, double strands breaks, EGF, epidermal growth factor, EGFR, epidermal growth factor receptor, ER, estrogen receptor, ER+ve, estreogen receptor positive, ERK, extracellular signal-regulated kinase, ESR1, estrogen receptor alpha gene, EXO1, exonuclease 1, FasL, fas ligand, FHRE, forkhead response element, Fox, forkhead box, GADD45, DNA damage-inducible protein 45, GICs, glioma-initiating cells, HCC, hepatocellular carcinoma, HR, homologous recombination, IGF, insulin growth factor, JNK/SAPK, c-Jun N-terminal kinase/stress activated protein kinase, MCL, mantle cell lymphoma, MDR1, multidrug resistance protein 1, MEK, mitogen-activated protein kinase kinase, miRs, Micro-RNAs, MMR, mismatch repair, MRN, MER11-RAD50-NBS1, Mst1, macrophage stimulating 1, mTOR, mammalian target of rapamycin, NAMPT, nicotinamide-phosphribosyltransferase, NBS1, Nijmegen breakage syndrome 1, NER, nucleotide excision repair, NHEJ, non-homologous end joining, OTUB1, OUT domain-containing ubiquitin aldehyde-binding protein 1, PA, Psammaplysene A, PDK, phosphoinositide-dependent kinase, PI3K, phosphatidylinositol 3-kinase, PIP3, phosphatidylinositol- 3,4,5-trisphosphate, PKB, protein kinase B, Rb2, retinoblastoma-related p130, ROS, reactive oxygen species, RTKs, receptor tyrosine kinases, SCs, stem cells, Skp2, S-phase kinase-associated protein 2, SOD-2, superoxide dismutase, Sox1, sex determining region Y box 2, SSBs, single-strand breaks, TCF/LEF, T-cell factor/lymphoid enhancer factor, TKIs, tyrosine kinase inhibitors, TRAIL, tumour necrosis factor-related apoptosis inducing ligand, VEGF, vascular endothelial growth factor, XRCC1, X-ray cross-complementing group 1, IFNɣ, interferon-ɣ

## Abstract

The FOXO3 and FOXM1 forkhead box transcription factors, functioning downstream of the essential PI3K-Akt, Ras-ERK and JNK/p38MAPK signalling cascades, are crucial for cell proliferation, differentiation, cell survival, senescence, DNA damage repair and cell cycle control. The development of resistance to both conventional and newly emerged molecularly targeted therapies is a major challenge confronting current cancer treatment in the clinic. Intriguingly, the mechanisms of resistance to ‘classical’ cytotoxic chemotherapeutics and to molecularly targeted therapies are invariably linked to deregulated signalling through the FOXO3 and FOXM1 transcription factors. This is owing to the involvement of FOXO3 and FOXM1 in the regulation of genes linked to crucial drug action-related cellular processes, including stem cell renewal, DNA repair, cell survival, drug efflux, and deregulated mitosis. A better understanding of the mechanisms regulating the FOXO3-FOXM1 axis, as well as their downstream transcriptional targets and functions, may render these proteins reliable and early diagnostic/prognostic factors as well as crucial therapeutic targets for cancer treatment and importantly, for overcoming chemotherapeutic drug resistance.

## 1 Introduction

Over the last decade, more than 100 members of Forkhead box (Fox) proteins have been identified in species ranging from yeast to human and grouped into FoxA to FoxR according to their characteristic ‘forkhead’ or ‘winged-helix’ DNA-binding domains and other functional sequences [1], [2]. These evolutionarily conserved Fox transcriptional regulators participate in a wide variety of cellular processes, including cell cycle progression, differentiation, proliferation, apoptosis, migration, metabolism, longevity, and DNA damage response (DDR) [3], [4]. Forkhead box O3 (FOXO3), previously known as FOXO3a and FKHR-L1, is a member of class ‘O’ subfamily of Forkhead box proteins (FOXOs). In humans, FOXO3, as well as the other three FOXO members (FOXO1, −4 and −6), bind to the same core consensus DNA sequence, the Forkhead Repsonse Element (FHRE; 5′-GTAAA(T/C)A-3′ or 5′-T(G/A)TTTAC-3′) in target genes to control transcription [5], [6]. In invertebrates, such as *Caenorhabditis elegans* and *Drosophila*, their genomes have only one FoxO gene, while mammals have four FOXO genes: FOXO1, FOXO3, FOXO4 and FOXO6 [7]. Given that these mammalian FOXO proteins recognize the same FHRE consensus, they can potentially regulate the expression of the same pool of target genes, which could lead to functional redundancy. Despite this, current evidence has supported the assertion that compared with other FOXO proteins, FOXO3 has a more predominant role in controlling cancer development and chemotherapeutic drug sensitivity [2]. This review will focus on the role and regulation of FOXO3 and its key downstream target FOXM1, and discuss their central roles as modulators of fundamental cellular processes with special emphasis on our understanding of the current therapeutic potential of targeting the FOXO3-FOXM1 axis in cancer.

## 2 FOXO3-FOXM1 axis: an attractive chemotherapeutic target

Multiple cellular proliferative signalling pathways converge on FOXO3 in the presence of different extracellular and intracellular stimuli. Most crucially, FOXO3 is negatively regulated by phosphatidylinositol 3-kinase (PI3K)-Akt/Protein Kinase B (PKB) and Ras-Raf-MEK-extracellular signal-regulated kinase (ERK) signalling pathways in reponse to a variety of external stimuli and growth factors [2], [8], [9]. Upstream of FOXO3, the PI3K-Akt and Ras-ERK pathways are activated by the binding of ligands, such as insulin growth factor (IGF) or epidermal growth factor (EGF), to their corresponding receptors. This induces the activation of cytosolic receptor protein tyrosine kinases, leading to tyrosine residues autophosphorylation. The phosphorylated residues become docking sites that recruit PI3K and subsequently generate a secondary messenger phosphatidylinositol- 3,4,5-trisphosphate (PIP3), which recruits phosphoinositide-dependent kinase (PDK) 1/2 to the plasma membrane, whereby PDK1/2 phosphorylates Akt [10]. The activated Akt then phosphorylates FOXO3 at Thr32, Ser253 and Ser294 and thereby provides binding sites for the chaperone protein 14-3-3 in the nucleus, which in turn, regulates FOXO3 transcriptional activity and cytoplasmic translocation. FOXO3 inactivation has frequently been identified in human tumours, largely due to the overactivation of the PI3K-Akt pathway [11], and this FOXO protein is thought to coordinate the balance between cell proliferation and cell death. Continuous FOXO3 inactivation triggers cells to shift towards proliferation and survival, and can eventually drive tumorigenesis with the additional activation of oncogenes or inactivation of other tumour suppressors [12]. Critically, the capability of FOXO3 to eliminate damaged or early transformed cells through cell death and senescence helps to establish FOXO3 as a *bona fide* tumour suppressor protein [8]. Similar to the PI3K-Akt pathway, external stimuli also activate Ras and in turn, induce the activation of subsequent kinases Raf, mitogen-activated protein kinase kinase (MEK) and extracellular signal-regulated kinase (ERK/MAPK) to phosphorylate FOXO3. Taken together, the Akt and ERK kinases negatively regulate FOXO3 activity directly by inducing a prompt and eventually sustained nuclear exclusion to the cytoplasm, where FOXO3 undergoes degradation and its activity curtailed [2], [13]. In addition, other extracellular and intracellular signals also contribute to affect FOXO3 localization and thus activity. For instance, oxidative stress results in FOXO3 phosphorylation by mediators or macrophage stimulating 1 (Mst1), which triggers FOXO3 and 14-3-3 dissociation and causes nuclear translocation of FOXO3, thus initiating the transcription of FOXO3 target genes [14].

**Fig. 1:**
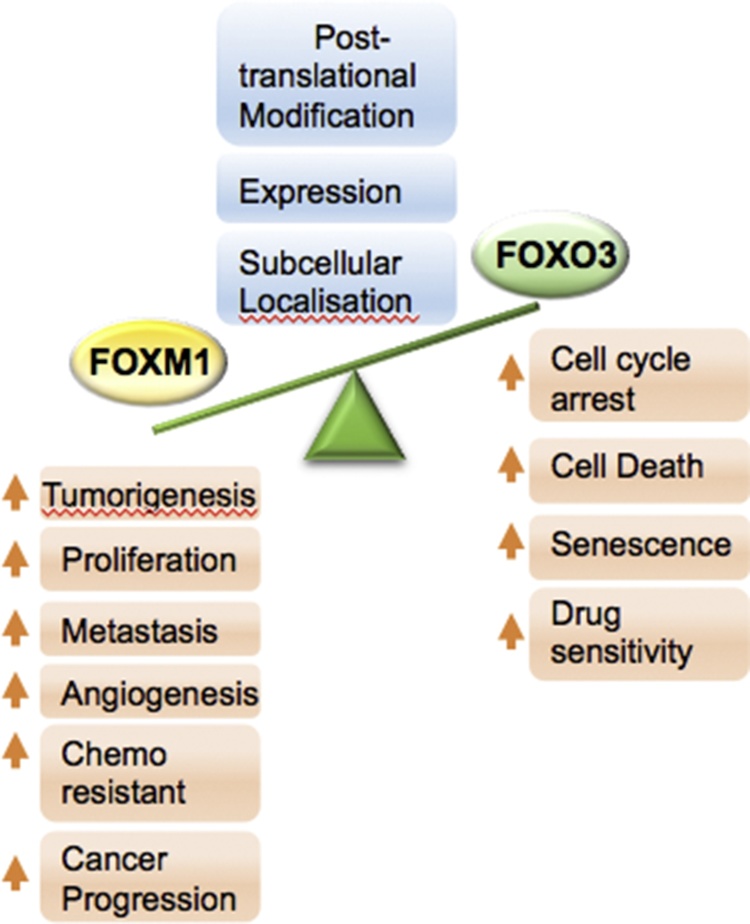
FOXM1 and FOXO3 direct cell fate in cancer. The potent oncogene FOXM1 antagonises the activity and expression of the tumour suppressor FOXO3 and vice versa. FOXM1 regulates a variety of biological processes important for cancer progression, including tumorigenesis, cell proliferation, metastasis, angiogenesis, and chemoresistance; while FOXO3 plays opposing roles by modulating cell cycle arrest, cell death, senescence and drug sensitivity. The regulation of FOXO3 and FOXM1 is through protein expression, post-translational medication and subcellular localisation.

**Fig. 2:**
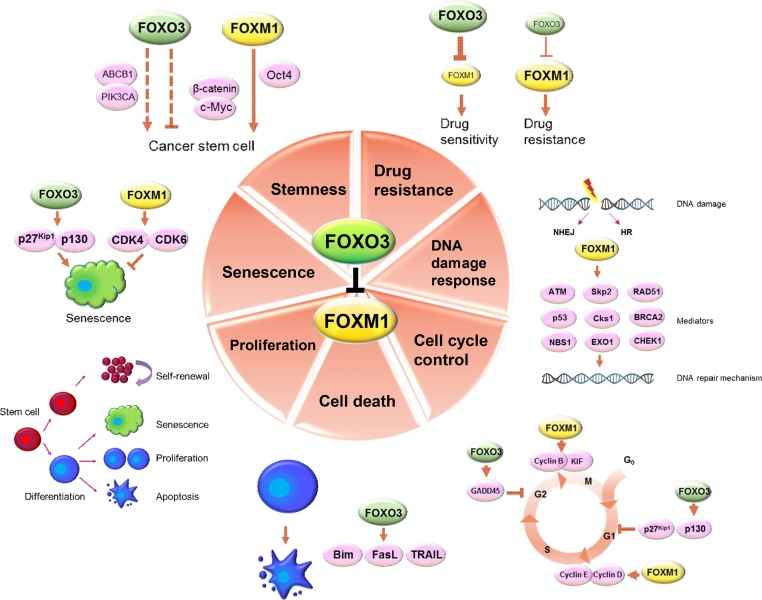
Functional roles of FOXO3 and FOXM1 in cancer drug resistance. FOXO3 antagonises FOXM1 activity and vice versa. FOXO3 and FOXM1 exert opposing functions in the regulation of cancer-related processes, including drug resistance, DNA damage response, cell cycle control, cell death, proliferation, cellular senescence and stemness, through the regulation of downstream gene targets.

A great number of FOXO3 downstream target genes have been identified and shown to participate in important cellular processes that govern cell fate [15], [16]. For instance, FOXO3 can initiate apoptosis by activating the transcription of pro-apoptotic genes B-cell lymphoma 2 interacting mediator (Bim), Fas ligand (FasL) and tumour necrosis factor-related apoptosis inducing ligand (TRAIL) [15], [16]. Furthermore, FOXO3 controls proteins involved in cell cycle and proliferative arrest. For example, FOXO3 upregulates the cell-cycle inhibitor p27^Kip1^ and the retinoblastoma-related p130 (Rb2) tumour suppressor to induce G0/G1 arrest and the DNA damage-inducible protein 45 (GADD45) to induce G2 arrest [15], [16]. Amongst the key downstream targets of FOXO3 involved in cell proliferation, cell survival and senescence, one of the most crucial targets is the potent oncogene FOXM1, whose expression and activity are negatively regulated by FOXO3 [2]. FOXO3 not only inactivates FOXM1 directly at the transcriptional level, but also antagonises FOXM1 function by competing for the same target genes [2], [17]. It has been shown that ectopic overexpression of FOXM1 can accelerate the development, proliferation, growth and survival of tumours in *in vivo* models [18]. In summary, FOXO3 activation leads to apoptosis, cell cycle arrest and FOXM1 repression in most tissues, whereas its inactivation favours enhanced cell survival, cell proliferation and FOXM1 expression (Fig. 1). As a consequence, a loss or gain of the function of the FOXO3-FOXM1 axis can alter cell fate. Specifically, tumorigenesis, cancer progression and the development of cancer therapy resistance have often been associated with FOXO3 inactivation or/and FOXM1 overexpression. This suggests that targeting the FOXO3-FOXM1 axis could be a viable strategy for the treatment of cancer. A better understanding of the mechanisms regulating the FOXO3-FOXM1 axis, as well as their downstream transcriptional targets, may render these proteins reliable diagnostic/prognostic factors as well as crucial therapeutic targets for cancer and for overcoming chemotherapeutic drug resistance (Fig. 2).

## 3 FOXO3-FOXM1: chemotherapy and resistance

Surgery is considered as the first-line treatment regime for most cancer patients, who are treated subsequently with conventional chemotherapy, radiation, hormonal therapy, and targeted therapy to reduce the risk of cancer recurrence in the adjuvant setting. Chemotherapies are also used if patients are inoperable and if surgery has not worked adequately. Anthracyclines (*e.g*. doxorubicin and epirubicin) and taxanes (*e.g*. paclitaxel and docetaxel) are the two conventional and most commonly used chemotherapeutic agents in the treatment of cancer, and are sometimes administered in a combinational manner [19], [20]. Anthracyclines function by inhibiting RNA and DNA synthesis and promoting ROS (Reactive Oxygen Species) formation which could damage DNA and proteins. Taxanes block cell cycle progression at mitosis by impairing centrosome formation, inducing abnormal spindles and preventing spindle-microtubule dynamics. These aberrant mitotic activities could trigger cell death, senescence and other anti-cancer effects [21]. The efficacy of these anti-cancer chemotherapeutic agents is often limited by the quick emergence of drug resistance. The mechanisms for the development of drug resistance can involve alterations in drug targets, drug metabolism, cancer stem cell population, DNA damage repair rate, as well as cell survival and death signals. Although these processes that contribute towards the development of drug resistance are different and diverse, they invariably integrate their activities and functions through the FOXO3-FOXM1 axis. For example, FOXM1 has been shown to be involved in mediating resistance to genotoxic agents, such as γ-irradiation and epirubicin, through regulating genes, including NBS1 and BRIP1, involved in DNA damage repair [22], [23]. Similarly, FOXM1 overexpression has also been demonstrated to confer acquired cisplatin resistance in breast cancer cells [24]. In agreement, overexpression of FOXM1 is a potential prognostic marker and enhances chemoresistance for docetaxel in gastric cancer [25]. Conversely, functional FOXO3 has been reported to determine the sensitivity of colon cancer cells to cisplatin [26]. In ovarian cancer, FOXM1 can also modulate cisplatin sensitivity by promoting the expression of the DNA damage-repair Exonuclease 1 (EXO1) [27]. Besides EXO1, FOXM1 also positively regulates a host of genes involved in DNA damage repair and cell cycle checkpoint control to overcome the cytotoxic and cytostatic effects of genotoxic agents [20]. These findings highlight that FOXM1 overactivation or FOXO3 inhibition can drive the expression of DNA damage response genes to increase repair or tolerance to DNA damage induced by genotoxic agents and thereby, contribute to the ability of cancer cells to survive the genotoxic function of anticancer drug. Paclitaxel can also downregulate FOXM1 to mediate mitotic catastrophe and senescence in breast cancer cells [28]. This cytotoxic function is at least in part mediated through the downregulation of the expression of the FOXM1 [28]. In concordance, overexpression of FOXM1 can enhance the resistance of breast cancer cells to paclitaxel and is a poor prognostic factor in breast cancer patients [28]. Recent results show aberrant Akt activation and FOXO3 inactivation confers 5-FU resistance [29], while the PI3K/mTOR inhibitor NVP-BEZ235 can effectively overcome 5-FU resistance through the up-regulation of PUMA expression, primarily through inactivation of PI3 K/Akt and activation of FOXO3, causing apoptosis even in cancer cells without functional p53 [29]. In addition to the more conventional chemotherapeutic drugs, the FOXO3 and FOXM1 transcription factors have emerged as the master regulators in novel molecular targeting anticancer agent resistance too. The pterocarpanquinone compound LQB-118 can induce apoptosis in drug-resistant myeloid leukaemic cells through specifically targeting FOXO3 and FOXM1[30]. Activation of FOXO3 has also been shown to reverse mitogen-activated protein/extracellular signal-regulated kinase kinase (MEK) 1/2 inhibitor AZD6244 chemoresistance in human cancer [31]. The p38α pharmacological inhibitior has also been shown to be able to combine with cisplatin to decrease colony formation and viability of cancer cells and strongly increase Bax-dependent apoptotic cell death by activating FOXO3 in colorectal cancer (CRC) cells[32]. Furthermore, overexpression of FOXM1 or downregulation of FOXO3 also reduces the sensitivity of EGFR/HER2 overexpressing breast cancer cells to tyrosine-kinase receptor inhibitors, such as gefitinib and lapatinib, while inhibition of FOXM1 or re-expression of FOXO3 restores sensitivity to these inhibitors in resistant breast cancer cells [33], [34].

## 4 Chemotherapeutic agents that target FOXO3-FOXM1

**Fig. 3:**
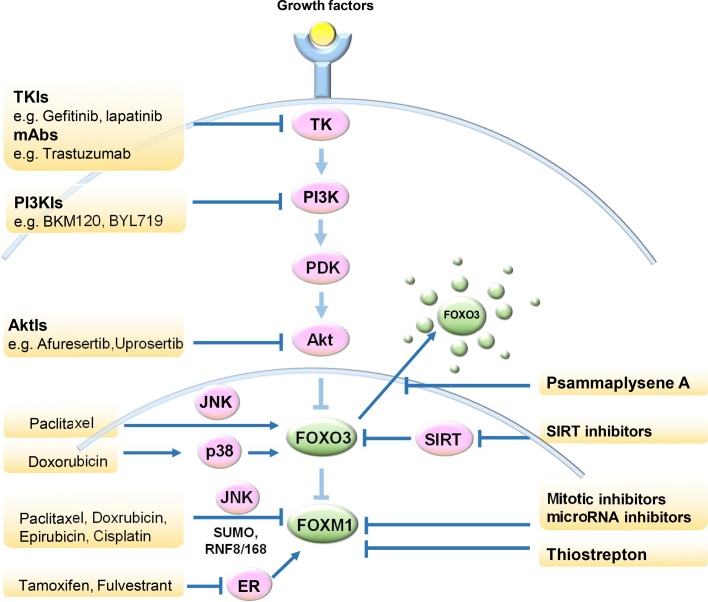
Integration of FOXO3-FOXM1 axis upon chemotherapeutic drugs. Current conventional and molecularly targeted chemotherapeutic agents as well as novel small molecule inhibitors have various modes of action but ultimately integrate their signals with the PI3K-AKT-FOXO3-FOXM1 signalling cascade.

The FOXO3-FOXM1 axis has a critical role in tumour suppression in terms of its function in cell cycle arrest, senescence, cell death and differentiation and other anti-cancer responses. Current anti-cancer drugs, including paclitaxel, doxorubicin, epirubicin, lapatinib, gefitibin, imatinib and cisplatin, have been confirmed to mediating their cytotoxic and cytostatic functions through FOXO3 and FOXM1. Paradoxically, dysregulation of the FOXO3-FOXM1 axis contributes towards drug resistance by modulating the expression of genes involved in drug efflux, DNA repair and cell survival in drug resistant cancers. Therefore, targeting FOXO3, FOXM1 and other upstream or downstream targets in the signalling pathways, could be a feasible strategy for cancer treatment and for overwhelming drug resistance. A range of studies have supported FOXO3 and FOXM1 as validated targets for drug development. Indeed, a number of conventional therapeutic agents and molecularly targeted drugs integrate their activities with FOXO3-FOXM1 to direct their anti-proliferative functions (Fig. 3).

### 4.1 Tyrosine kinase inhibitors (TKIs)

Typically, growth factor binding induces dimerization of receptor tyrosine kinases (RTKs), resulting in autophosphorylation that triggers downstream signals of multiple signalling pathways, including the PI3K-Akt and Ras-ERK cascades. The tyrosine kinases are thus key mediators of oncogenic signalling. For example, activation of EGF receptor (EGFR) and HER2 (also known as EGFR2) is an important factor for initiation and progression of malignancies, including breast and lung cancer. As a result, several tyrosine kinase inhibitors (TKIs) have been developed and confirmed to have effective anti-cancer activity. Some of these TKIs have also been approved by the Food and Drug Administration for use in patients, with many others coming into various stages of clinical trials. A number of approaches have been developed for blocking the deregulated EGFR/HER2-signalling pathway. Current TKIs, including gefitinib (Iressa), erlotinib (Tarceva), lapatinib (Tykerb), sunitinib (Sutent) and neratinib (Nerlynx), work by inhibiting the EGFR and/or HER2 signalling, thus preventing signals from being continuously activated and creating uncontrolled proliferation [35].

For example, gefitinib mainly inhibits tyrosine kinase activity of HER2, whereas, lapatinib targets the tyrosine kinase activity of both EGFR and HER2. Both TKIs have been shown to function through the FOXO3-FOXM1 axis. The EGFR-family of receptors are one of the most popular targets for anticancer therapies and many of them, such as erlotinib and gefitinib, have got enormous success in clinical treatments of cancer in the past decade. However, the efficacy of these agents is often hampered by the rapid emergence of drug resistance. In response to lapatinib treatment, FOXO3 is induced and nuclear translocated. Activated FOXO3 antagonises FOXM1 and thus competes for binding to its effectors, for example, vascular endothelial growth factor (VEGF), a key factor implicated with metastasis and angiogenesis in malignancies [17]. In addition, overexpression of FOXM1 reduces the sensitivity of HER2-positive breast cancer cells to lapatinib, while inhibition of FOXM1 rescues resistance to lapatinib resistance [33], [34]. Similarly, gefitinib attenuates EGFR signal transduction and is correlated with FOXM1 suppression [36]. This downregulation of FOXM1 is not seen in gefitinib resistant cells [36]. Taken together, FOXO3-FOXM1 axis has important functions in modulating TKI sensitivity (Table 1).

**Table 1:** Summary of FOXO3-mediated genes and functions.

Gene symbol	Functions	Type of malignancy	References
Bim, FasL, TRAIL	Apoptosis	Breast cancer, colorectal cancer, hepatocellular carcinoma (HCC)	[15], [16], [50]
p27^Kip1^, p130(Rb2), GADD45	Cell cycle and proliferative arrest	Breast cancer	[15], [16]
VEGF	Angiogenesis and metastasis	Breast cancer	[17]
FOXM1, miR-21, Fas-L, Survivin	DNA damage repair	Breast cancer	[46], [47]
ABCB1, TRAIL, SIRT1, 4, 5, 6, 7	Drug resistance	Breast cancer, colon cancer	[44], [50], [51], [52]
ABCB1, PIK3CA, β-catenin, c-Myc, MnSOD, SOD2	Cancer cell stemness	Brain glioblastoma, myeloid leukaemia	[89], [90], [91], [95], [121]
p27^Kip1^, p130(Rb2)	Senescence	Breast cancer	[128]
ATG, MAP1LC3, BNIP3,	Autophagy	Breast cancer	[135], [136]
VEGF	Tumour microenvironment	Breast cancer	[17]

Apart from these TKIs, other TK-targeted therapies such as monoclonal antibodies have also been reported with anti-cancer property, particularly when combined with the cytotoxic agents doxorubicin and paclitaxel [37]. These therapeutic monoclonal antibodies direct against the extracellular domain of EGFR or/and HER2. Trastuzumab (Herceptin) is the first clinically approved monoclonal antibody against HER2 mutant cancer [38], and its antiproliferative effects have also been shown to be mediated through FOXO3 and its downstream target Survivin (BRC5) *via* the PI3K-Akt pathway [39]. Other anti-HER2 monoclonal antibodies such as pertuzumab (Omnitarg), panitumumab (ABX-EGF) and Cetuximab (Erbitux) are under evaluation in clinical trials. In summary, these TKIs and monoclonal antibodies can inhibit the signalling transduction through the FOXO3-FOXM1 axis and thus prevent cancer cell proliferation. Further investigations into the role of the FOXO3-FOXM1 axis and development of TKIs and monoclonal antibodies will be required to maximize their efficacy.

### 4.2 PI3K and Akt inhibitors

As a major effector of RTKs, PI3K is targeted in pharmacological interventions to halt the RTK-PI3K-Akt signal transduction pathway. At present, a number of PI3K targeted compounds such as BKM120, BYL719, BEZ235, BGT226, XL 765, XL 147, GDC0941, SF1126, GSK1059615, PX-866 and CAL-101 are introduced into different stages of clinical trials, and some of which are dual PI3K/mTOR inhibitors [40]. The serine/threonine kinase Akt plays a critical role in integrating different proliferative and survival signalling pathways with the gene expression machinery. Akt directly inhibits FOXO3 to promote cell survival, cell cycle progression and proliferation. Akt overexpression is often associated with tumorigenesis, metastasis and resistance to chemotherapy or radiotherapy in cancer [41]. Therefore, Akt is also considered as an attractive target for cancer therapy. Despite the large number of compounds proven to inhibit Akt *in vitro* and *in vivo*, only a limited number of these, including AZD5363, afuresertib (GSK2110183), uprosertib (GSK2141795), ipatasertib (GDC-0068, RG7440), MK-2206 and TCN, have progressed to clinical evaluation [42]. For these PI3K and Akt inhibitors, FOXO3 has been shown to be the key downstream target. For example, in mantle cell lymphoma (MCL), PI3K and AKT, but not mammalian target of rapamycin (mTOR), inhibitors have been demonstrated to be able to cause significant reduction in leukaemic cell viability, which is again associated with FOXO3 nuclear translocation and activation [43]. In addition, as these PI3K and Akt inhibitors function upstream of the FOXO3-FOXM1 axis, they can also potentially integrate their anticancer activity with other chemotherapeutic drugs which also impact on FOXO3 and FOXM1 in combinatorial treatments for better therapeutic effects [42].

### 4.3 Genotoxic agents: regulation of DNA damage repair by FOXO3-FOXM1

Genotoxic agents, including doxorubicin, epirubicin, cisplatin and 5-FU, can cause DNA damage in forms of single-strand breaks (SSBs) and double-strand breaks (DSBs), which may result in detrimental mutations and in some cases, cancer. Cells have intrinsic mechanisms to prevent the accumulation of these harmful genotoxic mutations by either repairing the damaged DNA or undergoing apoptosis or senescence in DDR. The anticancer genotoxic agents are making use of this intrinsic DNA damage-induced cellular mechanism, DDR, to eliminate the relatively faster proliferating cancer cells. At the same time, some cancer cells can adapt and become resistant to the genotoxic agents by boosting their DNA repair mechanisms.

#### 4.3.1 Anthracyclines

These anthracycline derivatives are known as topoisomerase poisons that have the ability to inflict DNA damage and apoptosis by intercalating into the genome. Accumulating studies have demonstrated that these drugs are targeting FOXO3-FOXM1 axis to mediate their cytotoxic functions. Accordingly, dysregulation of FOXO3 and/or FOXM1 is responsible for the acquisition of multi-drug resistance in cancer cells [44]. The anthracycline doxorubicin induces FOXO3 activation and nuclear translocation by phosphorylating FOXO3 on Ser7 residue through p38 MAPK [45]. FOXO3 also mediates doxorubicin-induced apoptosis through its transcriptional repression of miR-21 which in turn, modulating expression of a pro-apoptotic factors, Fas-L, Bim and Survivin [46], [47]. Besides, FOXM1 has been shown as a downstream target of p38 MAPK, which is also downregulated in response to doxorubicin and epirubicin [48], [49]. Taken together, genotoxic agents induce FOXO3 activation and FOXM1 suppression to mediate their anti-proliferative function. The role of FOXO3 in doxorubicin resistance is complicated. It has also been shown that although FOXO3 initially triggering cell cycle arrest and apoptosis in response to doxorubicin, sustained FOXO3 activation promotes drug resistance and survival of cell by stimulating the membrane efflux transporter, ABCB1 expression [44]. Another study has further established that FOXO3 activation increases expression of TRAIL and cell death in response to doxorubicin in hepatocellular carcinoma (HCC), suggesting that FOXO3 is required for doxorubicin-sensitivity. On the other hand, inactivated or degraded FOXO3 in the cytoplasm is closely associated with doxorubicin resistance [50]. Furthermore, SIRT1 has been shown to deacetylate p53 and FOXO3 which contributes to inhibition of doxorubicin sensitivity [51]. Additionally, in breast cancer cells with acquired epirubicin resistance, SIRT 4, 5, 6 and 7 were found to be upregulated, particularly SIRT6, which has been shown to induce epirubicin resistance by mediating the deacetylation and inhibition of FOXO3 [52]. Similar mechanisms of action also apply to another anthracycline, epirubicin. Given that FOXM1 is the key factor in many aspects of DNA damage repair, it is involved in DDR by promoting DNA repair mechanism through regulating of multiple genes that essential for DDR. In general, FOXM1 protein level is higher in epirubicin resistant cells compared to sensitive breast cancer cells [53]. In the context of cancer chemotherapy, current study has shown that both p53 and apical kinases ataxia-telangiectasia mutated (ATM) regulate FOXM1 coordinately *via* E2F1 and consequently modulate epirubicin sensitivity in breast cancer [48], [53]. Overexpression of ATM is accompanied with high levels of FOXM1 has been observed in MCF-7 epirubicin resistance cells. In response to epirubicin, ATM is activated and subsequently induces E2F1 activity, E2F proteins enable FOXM1 expression and consequently enhance DNA repair and cell survival. Whereas p53 plays an opposing role in regulation of E2F1 by inhibiting its activity as well as reducing FOXM1 expression, which triggers cell death. Both ATM and p53 signals converge on E2F1 to regulate FOXM1 expression. The development of epirubicin resistance maybe due to the loss of p53 function and gain in ATM activities in the cell. Silencing ATM or FOXM1 has been found to re-sensitize the epirubicin resistant cells to epirubicin. In addition, FOXM1 has been shown to modulate DNA damage-induced senescence and thus epirubicin resistance through regulating Nijmegen breakage syndrome 1 (NBS1) expression [23]. Like FOXM1, overexpressed NSB1 has also been observed in MCF-7 epirubicin resistant cells and breast cancer patients with poorer outcome. NBS1 is a key component of the MER11-RAD50-NBS1 (MRN) complex, which has a central role in processing damaged DNA induced repair mechanism and in promoting ATM-dependent DDR signalling [54]. Depletion of FOXM1, accompanied by repressed NBS1 expression and reduced ATM activation, can render parental MCF-7 breast cancer cells and MCF-7 epirubicin resistant cells more sensitive to epirubicin-induced cellular senescence [23]. Furthermore, FOXM1 expression is regulated post-translationally in response to epirubicin treatment and these post-translational modifications are associated with epirubicin action and resistance in cancer cells. It has been shown that FOXM1 is SUMOylated in breast cancer cells in response to treatment with epirubicin and mitotic inhibitors, including paclitaxel [55]. Upon SUMOylation, FOXM1 becomes ubiquitinated and thereby, degraded by the ubiquitin–proteasome pathway in cytotoxic drug response. In this context, FOXM1-SUMOylation has a vital role in cytotoxic and genotoxic drug sensitivity. OTUB1 (OTU domain-containing ubiquitin aldehyde-binding protein 1) is a hydrolase that can specifically remove ‘Lys-48′-linked conjugated ubiquitin from proteins, and it positively regulates FOXM1 activity by promoting FOXM1 deubiquitination and stabilization [56]. Overexpression of OTUB1 and upregulation of FOXM1 expression are associated with enhanced proliferative rate and epirubicin resistance in breast cancer. Conversely, the ubiquitination E3-ligase RNF168 has been shown to cooperate with another E3 ubiquitin-protein ligase RNF8 to mediate FOXM1 ubiquitination and degradation in breast cancer response to epirubicin treatment [57]. Specifically, upon epirubicin treatment, the SUMOylated FOXM1 is recognized by RNF8, which recruits RNF168 to mediate further FOXM1 ubiquitination and degradation [57].

#### 4.3.2 Platinum compounds

Platinum Compounds, such as cisplatin, generate adducts on genomic DNA, which hamper DNA synthesis and repair, eventually inducing DNA damage and cytotoxicity in cancer cells. Several studies have documented FOXO3 as a major determinant in cisplatin sensitivity [26], [58]. Accordingly, it has been shown that cisplatin treatment causes a decrease in Akt-mediated FOXO3 phosphorylation in colon cancer cells, resulting in its nuclear translocation and activation. Conversely, impaired FOXO3 nuclear accumulation and the consequent failure to induce apoptosis have been associated with the development of cisplatin resistance. Interestingly, increased FOXO3 activation in cisplatin resistant colon cancer cells with a small molecule inhibitor, tricirbine/API-2, can reverse cisplatin resistance by blocking Akt [26]. Given the significant role of FOXM1 in DNA repair system, it is not surprising that enhanced FOXM1 corresponds to higher expression levels of DNA repair factors such as breast cancer- associated gene 2 (BRCA2) and X-ray cross-complementing group 1 (XRCC1) in cisplatin resistant cells compared with the sensitive MCF-7 cells. Silencing FOXM1 significantly can reduce the proliferative rate of cisplatin resistant cells, while overexpression of FOXM1 in sensitive cells enable to induce cisplatin resistance [24]. These findings establish a vital role of FOXO3 and FOXM1 in cisplatin sensitivity and resistance.

#### 4.3.3 DNA damage response in genotoxic agent action and resistance

Genotoxic anticancer agents, including doxorubicin, epirubicin and cisplatin, cause SSBs and DSBs. Cells can avoid harbouring genotoxic mutations by either repairing the damaged DNA or undergoing apoptosis. Homologous recombination (HR) and non-homologous end joining (NHEJ) are the two main mechanisms used by mammalian cells to repair DSBs. FOXM1 is involved in many aspects of the DDR and can thus contribute to drug resistance. FOXM1 has been shown to be essential for the repair of DSBs by HR [22], which is launched through the detection of DSBs by the MRN complex [59]. The MRN complex helps to recruit and activate key DDR signalling kinases, including ATM, and DNA repair proteins, at the sites of DNA damage. FOXM1 regulates the expression of NBS1 directly at the transcriptional level [52]. In consequence, FOXM1 can influence the initiation of HR, as the assembly of the MRN complex is rate-limiting for the recruitment and activation of ATM [60]. In addition, there is indication that the upregulation of NBS1 expression by FOXM1 also indirectly augments the stability of the other MRN components, MRE11 and RAD50, in the complex and thereby further amplifying the rate of HR repair [52]. Furthermore, FOXM1 can also promote HR repair indirectly through regulating the transcription of S-phase kinase-associated protein 2 (Skp2) and cyclin-dependent kinases regulatory subunit 1 (Cks1) [61], which are essential subunits of the Skp2-SCF E3 ligase complex that mediates the ubiquitination and thereby activation of NBS1. Consistently, Skp2 deficient cells display HR repair defects and are hypersensitive to ionizing irradiation [62]. Besides NBS1, FOXM1 also drives the transcription of a multitude of HR DNA damage repair genes, including EXO1, RAD51, XRCC1, BRCA2, CHEK1 (CHK1), and BRIP1 [22], [63], [64], [65]. During HR, the FOXM1 downstream target Exonuclease 1 (EXO1) is a multifunctional 5′ → 3′ exonuclease that helps to unwind duplex DNA to promote DNA end resection. Next, the breast cancer susceptibility gene product 1 (BRCA1), BRCA2 and several RAD51-related proteins promote the displacement of RPA by the strand exchange protein RAD51. RAD51 then searches for homologous sequences and catalyzes an exchange strand between the broken duplex and the intact sister chromatid. Furthermore, FOXM1 has also been suggested to be a transcriptional activator of BRCA2 [63], a vital HR protein which binds the ssDNA and recruits the recombinase RAD51 to stimulate strand invasion during HR. Appropriately, induction of RAD51 by FOXM1 in glioblastomas has been shown to confer resistance to the genotoxic alkylating agent temozolomide [63]. In addition, the FOXM1 target BRIP1 also binds to and functions cooperatively with BRCA1 to promote HR repair. Bound BRIP1 unwinds damaged dsDNA to allow other repair proteins to access and process the damaged DNA [22], [66]. FOXM1 has also been shown to drive the transcription of CHK1 to induce cell cycle checkpoints to allow HR repair to proceed [65], and appropriately, CHK1 inhibition potentiates the therapeutic efficacy of radiotherapy [67].

The majority of DSBs occurring throughout the cell cycle are repaired by NHEJ. Although FOXM1 has been shown to be dispensible for DNA repair by NHEJ but not HR [22], FOXM1 still play an integral part in NHEJ. For example, the MRN complex is also required for processing and repairing etoposide- and anthracyclin-induced DSBs by NHEJ in G0/G1 phase of the cell cycle [68]. Appropriately, cells deficient in MRE11 or NBS1, but not ATM, exhibit a severe NHEJ repair defect, suggesting a key role of MRN in NHEJ repair independent of ATM [68]. The multifunctional FOXM1-regulated EXO1exonuclease is again involved DDB resection during NHEJ in G1 phase of the cell cycle [69]

FOXM1 also participates in the three major cellular mechanisms primarily involved in the repair of SSBs: nucleotide excision repair (NER), base excision repair (BER), and mismatch repair (MMR). For example, in NER, FOXM1 has been shown to regulate the expression of PolE2, RFC4 and RFC5 at the transcriptional level. The PolE gene encodes for DNA pol ε which plays a role in NER-dependent DNA synthesis, while RFC4 and –5 are subunits of RFC, which cooperates with PCNA during NER [27], [70]. In addition, FOXM1 is further linked to NER through the transcriptional regulation of RAD23B, a cofactor involved in the initiation of NER [71]. FOXM1 can also influence NER in quiescent cells *via* controlling the expression of XRCC1, [71]. In addition, FOXM1 also directly regulates the transcription of BRCA1-interacting protein-terminal helicase 1 (BRIP1; also called BACH1 and FRACJ), which processes interstrand crosslinks during MMR. This is mediated, in a BRCA1 independent manner, through its interaction with the MutLα mismatch repair complex, [72]. FOXM1 can also promote a number of SSB repair mechanisms, including NER, MMR and BER, by transcriptionally activating the expression of ssDNA repair genes, such as *RFC4*, *EXO1*, and *PolE2* [27], [71]. During ssDNA repair, the gap created by 5′–3′ nuclease activity of EXO1 is filled with the correct base by DNA pol δ and ε and the remaining nick rejoined by DNA ligase. This repair process is again orchestrated by the RFC and PCNA that loads and clamps DNA pol, for DNA synthesis. The significance of this regulation of Exo1 by FOXM1 in DDR is highlighted by the revelation that FOXM1 modulates the sensitivity to the DNA-damaging agents, cisplatin and doxorubicin, through regulating EXO1 in ovarian and breast cancer, respectively [27], [73]. In addition, FOXM1 has been shown to regulate the expression of the DNA methyltransferase DNMT1 through the chromatin remodelling factor HELLS [74]. Intriguingly, DNMT1 has a novel methyltransferase-independent role in promoting DNA damage repair through decondensing chromatin local to sites of DNA damage [75]. Collectively, these findings provide compelling evidence that FOXM1 plays an integral part in DDR through driving the transcription of genes encoding for DNA damage sensors, mediators, signal transducers and effectors to enhance the rate of DDR.

### 4.4 Mitosis modulators

Paclitaxel functions by inducing microtubule dysfunction and eventually cell death [28], [47], [76], [77]. Paclitaxel has been shown to enhance the activity of c-Jun N-terminal kinase/stress activated protein kinase (JNK/SAPK) which in turn, phosphorylates and activates FOXO3 directly, as well as repressing the inhibition of Akt on FOXO3 [47], [77], [78]. The activated FOXO3 then activates pro-apoptotic molecules, such as Bim, to trigger apoptosis [47], [77]. Moreover, downstream of FOXO3, FOXM1 also plays a key part in modulating paclitaxel action and sensitivity. Paclitaxel has been shown to downregulate FOXM1 to mediate mitotic catastrophe and breast cancer paclitaxel sensitivity. Consistently, FOXM1 depletion can sensitize breast cancer to paclitaxel-induced senescence [28]. FOXM1 regulates the expression of the microtubulin-associated kinesin KIF20A at the transcriptional level [28]. FOXM1 and KIF20A depletion can similarly promote abnormal mitotic spindle morphology and chromosome alignment, which have been shown to induce mitotic catastrophe-dependent senescence, suggesting that paclitaxel represses the FOXM1-KIF20A axis to drive abnormal mitotic spindle formation and mitotic catastrophe and that deregulated FOXM1 and KIF20A overexpression may confer paclitaxel resistance [28]. Appropriately, FOXM1 overexpression in ovarian cancer correlates with poor patient survival and contributes to the development of paclitaxel resistance [79].

### 4.5 Endocrine therapy

The estrogen receptor (ER) pathway plays a pivotal role in breast cancer development and progression. Endocrine therapy is highly effective in blocking the ER pathway, but its usefulness is limited by common intrinsic and acquired resistance. For example, Tamoxinfen and Fulvestrant are endocrine therapeutics commonly used in the management of estrogen receptor positive (ER+ve) breast cancers by blocking ER signalling. Recent studies have shown that the ERα gene (ESR1) is a transcriptional target of FOXM1 [80], and conversely, FOXM1 also regulates the expression of ERα at the transcriptional levels [81]. In concordance, there is a positive correlation between ERα and FOXM1, and an inverse correlation between ERα and FOXO3 in breast cancer patients. It has also been shown that in ER+ve breast cancer cells, tamoxinfen and fulvestrant treatment represses FOXM1 expression [82]. This positive feedback loop might modulate hormonal therapy sensitivity, and its deregulation or uncoupling might therefore have implications for endocrine sensitivity [80]. Furthermore, overexpression of FOXM1 can confer resistance towards tamoxinfen and fulvestrant in endocrine sensitive ER+ve breast cancer cells [80]. Consistently, in breast cancer patients with ER+ve tumours, low FOXM1 expression is associated with better survival [83]. In addition, recent genome-wide mapping of FOXM1 binding by chromatin immunoprecipitation with massively parallel DNA sequencing (ChIP-seq) analysis reveals the co-binding of FOXM1 with ERα in breast cancer cells, suggesting that FOXM1 is a common co-factor of ERα [84]. This indicates that an intimate functional relationship between FOXM1 and ERα in breast cancer development and probably, endocrine therapy sensitivity. ERα has antiproliferative and proapoptotic functions in breast cancer. Consistently, ERβ1 has been shown to repress FOXM1 expression through targeting ERα to control cell proliferation in breast cancer [85]. Particularly, ERβ1 represses FOXM1 transcription through competing with ERα to bind to an estrogen-response element located within the proximal promoter region of FOXM1.

### 4.6 Cancer stem cells: a renewal source of chemoresistant cells

Cancer stem cells (CSCs) or tumour-initiating cells, are a small population of cancer cells that have tumorigenic ability and share some common characteristics with normal stem cells (SCs) [86]. Besides cancer metastasis and relapse, CSCs are also profoundly involved in drug resistance. There is compelling evidence that FOXO3 may affect the development of the CSCs. The activation of the PI3K-mTOR-STAT3 pathway has been shown to be required for the viability and maintenance of breast cancer stem cells. Conversely, overexpression of PTEN, the antagonist of PI3K activity, decreases breast cancer cell tumorigenicity [87]. PTEN negatively regulates the PI3K-Akt signalling pathway and thus restores FOXO3 activity in the nucleus. It has been shown that FOXO3 nuclear translocation inhibits CSC maintenance in prostate cancer. Consistently, FOXO3 deficiency (*foxo3^−/−^*) leads to expansion of the CSC pool as well as increased self-renewal and tumorigenic capacity [88]. In addition, FOXO3 has also been shown to have a ‘negative’ role in CSC maintenance [89]. Accordingly, the combined inhibition of PI3K/Akt and Ras/ERK pathways which converge on FOXO3 in brain glioblastoma CSCs could block the self-renewal and tumorigenicity of CSCs [89]. Paradoxically, adverse prognostic impact of high FOXO3 expression in acute myeloid leukaemia (AML) patients has also been documented [90]. This is in agreement with research which has shown that nuclear localization of FOXO3 is required for CSC survival in chronic myeloid leukaemia (CML) [91]. Indeed, FOXO3 has been shown to positively regulate the expression of ABCB1 and PIK3CA, two stem cell associated drug-resistant genes, in a feedback mechanism. ATP- binding cassette sub-family B member 1 (ABCB1), also known as P-glycoprotein 1 and multidrug resistance protein 1 (MDR1), is responsible for the efflux of anthracyclines and taxanes in cancer cells [92]. PIK3CA encodes for the catalytic p110 subunit of the PI3K and its induction can potentially augment the pro-survival PI3K-AKT signalling. These findings could indicate a ‘positive’ role for FOXO3 in the maintenance of CSCs or that FOXO3 can be deregulated and consequently, express at high levels without any adverse proliferative effects on CSCs. The contrasting roles of FOXO3 in the maintenance of CSC properties documented may suggest that FOXO3 may have different functions in CSCs of different cancer types. Hitherto, it remains totally unclear what causes these contrasting FOXO3 functions among different cancers, but the identification of FOXO3 modifications, co-factors and downstream targets involved in the control of different CSCs might provide informative clues. This will also have important implications in the development of agents that target CSCs for improved treatment of specific cancer.

Salinomycin is an ionophore antibiotics which can selectively eliminate CSCs [93], [94]. In HCC cells, salinomycin has been shown to function through activating FOXO3 to disturb the β-catenin and TCF complex and inhibiting the expression of β-catenin/TCF target genes (eg. ZEB1, c-Myc and Cyclin D1), involved in CSC development [95]. Similarly, the anti-cancer small molecule ONC201/TIC10 has also been shown to inhibit colorectal CSC self-renewal *via* inhibiting Akt and ERK, and consequently activating FOXO3 to induce the expression of the death receptors TRAIL and DR5 (TRAIL-RII) [96]. Recent evidence further suggests that blockage of CXCR1-mediated cell signalling using either antibodies or the small-molecule inhibitor repertaxin induces the pro-apoptotic FAS-Ligand production to deplete breast CSCs *via* the FAK/AKT/FOXO3 signalling pathway [97]. Downstream of FOXO3, FOXM1 has also been demonstrated to promote CSC phenotypes [98], [99], [100]. Accordingly, FOXM1 is required for maintaining pluripotency and self-renewal ability of embryonal carcinoma cells [101]. FOXM1 has also been found to promote the proliferation and maintenance of pancreatic, breast and lung cancer stem cells [99], [102], [103], [104], [105], and its depletion represses the stemness of these cancer cells. Oct4, Sox2 and c-Myc are key factors involved in the reprogramming of cancer cells into cancer stem cells [106], [107]. FOXM1 has been shown to promote stem cell pluripotency by promoting the transcription of Oct4 (also called K15, POU5F1), which helps to suppress cellular differentiation [101]. In addition, FOXM1 can bind to the promoter of the c-myc gene and transactivate its expression [102], [108], [109]. On the other hand, FOXO3 can repress c-Myc activity, while FOXM1 is also a target gene of c-Myc [11], a transcription factor involves in the maintenance of the self-renewing multipotent stem cell population responsible for the regenerative capacity of tissues, such as the mammary epithelium [110]. Furthermore, FOXM1 has also been shown to regulate the expression of the pluripotency genes sex determining region Y box 2 (Sox2) and Bmi1 to promote stem cell-like properties [111], [112]. FOXM1 can also indirectly influence stem cell maintenance by interacting with β-catenin and inducing its nuclear translocation and transcription activation [113], [114]. The nuclear relocated β-catenin then complexes with T-cell factor/lymphoid enhancer factor (TCF/LEF) to trigger the transcription of Wnt targets which are required for the promotion of stem cell phenotypes in cancer cells [113], [114], [115]. In addition, FOXM1 is also required for β-catenin-mediated mammary stem cell amplification and tumorigenesis [104], [105]. In summary, FOXM1 is an important component of the reprogramming network and function together with Oct4, Sox2 and Myc to regulate CSC self-renewal and maintenance.

#### 4.6.1 ROS in the regulation of FOXO3 in CSCs

ROS are the byproducts of cellular aerobic metabolism, and have been found to be elevated in many cancer types [116]. Augmented endogenous ROS lead to adaptive cellular adjustments to control ROS levels, which may have a role in tumorigenesis, metastasis, angiogenesis and resistance to radiation and chemotherapy [117]. Conversely, the ROS generated by chemotherapeutic agents may disturb the redox balance and selectively eliminate cancer cells but spare normal cells. Normal stem cells and CSCs are characterized by low cellular ROS, which are critical for maintaining the potential for self-renewal and stemness [116]. ROS have been shown to have a role in the development and maintenance of CSCs, and this function is also closely linked to FOXO3. In agreement, ROS-mediated activation of p38 MAPK and FOXO3 has been shown to have a pivotal role in the induction of differentiation and the repression of tumour-initiating capacity of human glioblastoma-derived glioma-initiating cells (GICs) [118]. Specifically, ROS triggered p38-dependent BMI1 protein degradation and FOXO3 activation in GICs. Indeed, FOXO3 phosphorylation by p38/JNK MAPK on Ser-7 has been shown to result in its nuclear localisation and activation as well as its acetylation by CBP/p300 on Lys-242/245 [119]. In addition, FOXM1, which is negatively regulated by FOXO3, can block ROS-induced cellular senescence by stimulating the expression of the self-renewal gene BMI1 [112].

The ability of CSCs to maintain low cellular ROS levels is intimately linked to increased expression of free radical scavenging proteins [120]. Interestingly, FOXO3 can also protect cells against ROS *via* the upregulation of MnSOD and superoxide dismutase (SOD2), two scavenger proteins that are involved in the detoxification of ROS [121]. MicroRNAs (miRs) have emerged as an important class of cellular regulators of CSCs. In breast cancer, [122] miR-221-222 targets FOXO3 [123] and is involved in the promotion of an aggressive breast CSC-related phenotype and EMT [124], [125]. Consistently, the miR-221 and −222 are upregulated in the human breast, pancreatic and glioblastoma CSCs [122], [123], [126], [127].

#### 4.6.2 Senescence

Senescence is a permanent state of cell cycle arrest, and the age-dependent decline of the renewal capacity of stem cells. The cancer stem cells divide indefinitely, while other cancer cells eventually undergo senescent and stop dividing. FOXO3 overexpression can induce cells to enter senescence through promoting the expression of p27^Kip1^ [128]. In addition, activation of FOXO3 also induces the expression of the retinoblastoma family protein p130 (RB2) to trigger senescence in cycling cells. There is strong evidence that FOXM1 has a crucial role in promoting stem cell renewal by suppressing senescence. FOXM1 can counteract oxidative stress-induced senescence through enhancing Bmi-1 transcription [112]. Moreover, FOXM1 is overexpressed in gastric cancer, and its inhibition leads to cellular senescence [129]. In agreement, overexpression of the tumour suppressor microRNA miR-506 can induce senescence in ovarian cancer cells through repressing CDK4 and −6 expression and its activation of FOXM1 [130]. Similarly, the CDK4/6 inhibitor LEE011 can also induce senescence in neuroblastoma cells through targeting the induction of FOXM1 by CDK4/6 [131]. Another microRNA, miR-370, is often downregulated in AML, and reinstate of its expression curtails FOXM1 expression, induces cellular senescence in AML cells [132]. Collectively, these findings confirm a role of FOXM1 in suppressing cellular senescence in cancer cells.

### 4.7 Autophagy

Autophagy is an evolutionarily conserved cellular ‘self-digesting’ process which can be further divided into three types: macro-autophagy, micro-autophagy and chaperone-mediated autophagy. Autophagy in cancer cells is used to increase stress tolerance in cells, thus promoting tumour cell survival. Recent evidence have shown that FOXO1 and FOXO3 are involved in the modulation of autophagy, especially in response to oxidative stress [133]. In cancer cells, in response to oxidative stress or serum-starvation, FOXO1 can be acetylated by Sirtuin-2 (Sirt-2), where the acetylated FOXO1 can bind to Atg-7 thereby mediating transcription-independent autophagy and promoting tumour suppression [134]. On the other hand, in skeletal muscle cells, FOXO3 increases autophagy by directly up-regulating the transcription of autophagy-related (ATG) genes or autophagy regulatory genes, including microtubules-associated protein 1 light chain (MAP1LC3), BCL2/adenovirus E1B19-kDa interacting protein 3 (BNIP3) [135], [136]. In fact, FOXO3 can induce transcription-dependent autophagy through upregulation of class I PI3K/Akt activity in a FOXO1-dependent manner. The underlying mechanism is that FOXO3 can upregulate the class I PI3k/Akt activity, which in turn phosphorylate and induce nuclear exportation of FOXO1, as a result promoting autophagy. In addition, FOXO3 also co-ordinately upregulates FOXO1 by binding to its promoter region, thus upregulating FOXO1 transcription, to induce autophagy [137]. In short, inhibition of autophagy in tumours with high levels of autophagy through targeting FOXO3-FOXM1 axis may be an efficient approach for developing new successful treatments.

### 4.8 Tumour microenvironment (TME)

Tumour microenvironment (TME) consists of various components that have a major role in influencing the metabolic phenotypes of tumour cells and tumour progression. It provides support and protection for tumour growth, as well as playing critical roles in developing drug resistance. The TME components include surrounding immune cells, blood vessels and the extracellular matrix (ECM). It is found that FOXO3 represses the vascular endothelial growth factor (VEGF) by binding to its transcriptional promoter region, whereas FOXM1 induces VEGF expression [17]. VEGF is an important growth factor in developing new blood vessels surrounding tumour cells in which supplying nutrients and oxygen for tumour growth. Taken together, overexpressed FOXM1 induces VEGF secretion in TME and thus triggering nutrients supply to the tumour cells; on the other hand, increased levels of FOXM1 are closely associated with the development of chemotherapeutic resistance, which further promotes tumour growth and survival. TME also promotes immunological tolerance, guarding tumour from immune cells detection. The plasmacytoid dendritic cells (pDCs), which has a dual role not only in initiating immune responses but also in enhancing tolerance. FOXO3 has been shown to be a novel regulator of the immunological tolerance associated pDCs, and thereby, promotes the immunosuppressive activity of tumour-associated pDCs (TApDCs) [138]. Consistently, high expression of FOXO3 blocks regulatory signalling pathway of TApDCs in mouse models [138]. Furthermore, FOXO3 and FOXO1 have been shown as critical regulators of CD8+ T cell memory by inhibiting T-bet expression and thus the CD8+ T cell effector molecules interferon-ɣ (IFNɣ) and granzyme B [139], [140]. Therefore, high levels of FOXO3 expression also reduce long-lived CD8+ T cell survival.

## 5 Future perspectives and conclusion

### 5.1 Future perspectives: targeting the FOXO3-FOXM1 axis

The rationale of developing novel, effective and targeted therapies relies on the availability of knowledge of molecular mechanisms involved in carcinogenesis, cancer progression and drug action. In the case of cancer drug resistance, an understanding of the upstream regulators, downstream targets and cellular functions of FOXO3-FOXM1 in cancer can provide us with clues to design novel therapeutic strategies to eliminate cancer and to override drug resistance through targeting FOXO3 and FOXM1 (Fig. 3). FOXO3 expression and activity is extensively regulated by post-translational modifications, including phosphorylation, acetylation and ubiquitination [141]. Given that FOXO3 is a direct downstream target of Akt(PKB), the reactivation of FOXO3 is a key function of Akt inhibitors, particularly in cancers with overactivation of the RTK(receptor thyrosine kinase)-PI3K-Akt axis. Consistently, it has been reported that the Akt inhibitor, OSU-03012, can induce FOXO3 dephosphorylation levels and nuclear relocation in breast cancer cells [142]. Another potential means to enhance the activity of DNA damaging agents and overcome genotoxic resistance is through inhibiting the Class III NAD-dependent deacetylases, Sirtuins (SIRT1-7) [143], which are key regulators of FOXO3 deacetylation and expression [51], [52]. Compelling evidence has shown that SIRT1 and SIRT2 deacetylate FOXO3 and target it for proteasomal degradation [144]. Inhibitors against individual as well as a number of SIRTs have already been developed, and some have demonstrated anticancer activities, through targeting tumour suppressors [144], [145] including p53 and FOXO3. FOXO3 can be regulated by SIRT1 in cancer cells under oxidative stress conditions where FOXO3 is highly acetylated [51]. Nuclear SIRT1 can deacetylate FOXO3, resulting in enhanced expression of the DNA repair *Gadd45a* gene but decreased expression of pro-apoptotic targets such as FasL and Bim [51], supporting the idea that SIRT1 promotes FOXO3-mediated genotoxic drug resistance and limits cell death. In agreement, it has also been reported that the FOXO3-mediated induction of Gadd45a expression can be negatively regulated by nicotinamide-phosphoribosyltransferase (NAMPT), a stress-induced protein, and SIRT1 [146]. Chemical inhibition of NAMPT or SIRT1 can result in increased FOXO3 acetylation and activity, and thereby, Gadd45a upregulation. Another nuclear Sirtuin, SIRT6, has recently been shown to modulate FOXO3 expression [52]. In the context of DNA damage repair, SIRT6 can promote epirubicin resistance, while cells lacking SIRT6 sustain higher levels of DNA damage in response to epirubicin and γ-irradiation, suggesting that SIRT6 deletion results in inefficient DNA damage repair in response to DNA-damaging agents. Importantly, these effects of SIRT6 on DNA repair and drug resistance appear to be mediated, at least in part, through FOXO3 deacetylation by SIRT6 [52]. Consistent with this, SIRT-inhibitors, including Sirtinol, Salermide, and EX527, have been shown to be able to have anti-cancer activity and can combine with DNA damaging agents, such as doxorubicin, to eliminate breast cancer cells [147]. Besides these agents, The bromotyrosine derivative Psammaplysene A (PA) has been identified to be able to increase FOXO3 nuclear localization and hence promoting the transcription of its target genes, while repressing FOXO3 degradation in cytoplasm [148]. FOXO3 antagonises FOXM1 by competing for the same gene targets and thus playing opposing functions in tumorigenesis, metastasis and drug sensitivity. As a result, inhibition of FOXM1 could be another obvious strategy in anti-cancer treatment. The thiazole antibiotic thiostrepton has been shown to directly target FOXM1 and inhibit its binding to the promoter/enhancer regions of cell cycle regulatory genes [149], [150]. Breast cancer cells treated with thiostrepton have been shown to induce cell cycle arrest and cell death, and lead to repression of cancer cell migration, metastasis and transformation [149]. In addition, SUMOylation modulators have attracted more and more attentions because of their great potentials as future therapeutics [151], [152]. Both SUMOylation and FOXM1 are essential in cell cycle transition. Studies have shown that SUMOylation attenuates FOXM1 activity and triggers mitotic delay in breast cancer cells [153]. As a consequence, there is no doubt that SUMOylation modulators might be a potential interesting addition to the anti-cancer therapeutic arsenal targeting FOXM1. Furthermore, p300/CBP and the miR-320 microRNA have also been reported to repress FOXM1 activity directly and therefore employed to restrict cancer cell proliferation [154], [155], providing another strategy for intervention. In addition, it has also been shown that Aurora kinase A (AURKA) can cooperate with FOXM1 in a tightly coupled positive feedback loop, which is required for the self-renewal of breast cancer stem cells [156]. Specifically, nuclear AURKA is recruited by FOXM1 as a co-factor to transactivate FOXM1 target genes in a kinase-independent manner. In this context, targeting the AURKA-FOXM1 axis using the novel small molecule AURKA inhibitor AKI603 may represent a novel therapeutic strategy that can be applicable for FOXM1 overexpressing drug-resistant tumours. Consistently, the AURKA (AKI603) and FOXM1 (thiostrepton) inhibitors, also function synergistically to suppress proliferation and self-renewal of breast CSCs [156].

Recent immunohistochemical analysis has revealed that phosphorylated adenosine monophosphate-activated protein kinase (AMPK) at Thr172 (the active form of AMPK) is highly expressed in normal breast epithelial tissues compared with primary breast cancer samples [157]. AMPK is a highly conserved energy sensing serine/threonine kinase that regulates a number of physiological process and mediates metabolic reprogramming to thrive against nutrient deprivation by promoting catabolism (fatty acid oxidation, autophagy and glycolysis) and suppressing anabolism (protein, cholesterol and fatty acid synthesis) [158]. Interestingly, *in vitro* kinase assays has showed that AMPK can phosphorylate and activate FOXO3 directly on residues Thr179, Ser399, Ser413, Ser439, Ser555, and Ser588 in an AMP-dependent manner [159], which in turn promotes FOXO3 protein nuclear localization and transcriptional activities. In times of nutrient deprivation and oxidative stress, AMPK can activate FOXO3 directly *via* targeted phosphorylation. The interactions between AMPK signalling pathway and FOXO proteins suggest that the decline in AMPK activity is closely related to the development and progression of cancer. In agreement, a study on aggressive, invasive and drug resistant MDA-MB-231 breast cancer cell line has showed that metformin, a glucose lowering drug, has strong anti-proliferative effects on MDA-MB-231 cells through an AMPK-dependent pathway even in the absence of LKB1, an activator of AMPK, and also *via* p27Kip1 and p21Cip1, which are known transcriptional targets of FOXO3 [160]. In agreement, a novel AMPK activator, OSU-53, has also been shown to directly stimulate activity of AMPK and inhibit the viability and clonogenicity of MDA-MB-231 and MDA-MB-468 breast cancer cells. OSU-53 also suppresses fatty acid biosynthesis and shifts the metabolism to oxidation, suggesting the anti-tumour effects of OSU-53 in breast cancer cell lines might be due to its ability to modulate energy homeostasis in an AMPK-dependent manner [161]. More importantly, another group has reported that OSU-53, metformin and AICAR treatments can blunt the mesenchymal properties of breast and prostate cancer cells and the phenotypic changes are due to AMPK-mediated FOXO3 activation [162], further supporting the importance of AMPK-FOXO3 signalling axis in the progression and metastasis of breast cancer as well as other types of cancers. All in all, targeting the AMPK-FOXO3 signalling axis and lipid metabolism can therefore be a relevant therapeutic strategy in treating breast cancers, in particular drug resistant breast cancers.

### Conclusion

In summary, our review explores the fundamental principles as well as the most up-to-date knowledge on the FOXO3-FOXM1 axis. FOXO3 and FOXM1 have emerged as crucial factors in various aspects key to cancer progression, and they include cell cycle arrest, senescence, self-renewal, metabolism, drug sensitivity and resistance. The complexity of signal transduction that converges on the FOXO3-FOXM1 axis which involves negative feedback loops and extensive cross-talks with other signalling pathways, provides extensive opportunities and adaptive pathways for cancer cells to develop resistance to conventional and novel targeted therapeutics. A better understanding of the FOXO3-FOXM1 axis, the mechanisms for drug action and those responsible for the development of chemoresistance will undoubtedly assist in designing combination and sequential treatments to improve patient outcomes in the clinic.

## Conflict of interest

The authors declare no conflict of interest.
